# Bed-Based Ballistocardiography: Dataset and Ability to Track Cardiovascular Parameters

**DOI:** 10.3390/s21010156

**Published:** 2020-12-29

**Authors:** Charles Carlson, Vanessa-Rose Turpin, Ahmad Suliman, Carl Ade, Steve Warren, David E. Thompson

**Affiliations:** 1 Mike Wiegers Department of Electrical and Computer Engineering, Kansas State University, Manhattan, KS 66506, USA; suliman@ksu.edu (A.S.); swarren@ksu.edu (S.W.); davet@ksu.edu (D.E.T.); 2 Department of Kinesiology, Kansas State University, Manhattan, KS 66506, USA; vanessaturpin@ksu.edu (V.-R.T.); cade@ksu.edu (C.A.)

**Keywords:** ballistocardiography, unobtrusive cardiac monitoring, shared biomedical database, cuff-less blood pressure monitoring, force sensors

## Abstract

Background: The goal of this work was to create a sharable dataset of heart-driven signals, including ballistocardiograms (BCGs) and time-aligned electrocardiograms (ECGs), photoplethysmograms (PPGs), and blood pressure waveforms. Methods: A custom, bed-based ballistocardiographic system is described in detail. Affiliated cardiopulmonary signals are acquired using a GE Datex CardioCap 5 patient monitor (which collects ECG and PPG data) and a Finapres Medical Systems Finometer PRO (which provides continuous reconstructed brachial artery pressure waveforms and derived cardiovascular parameters). Results: Data were collected from 40 participants, 4 of whom had been or were currently diagnosed with a heart condition at the time they enrolled in the study. An investigation revealed that features extracted from a BCG could be used to track changes in systolic blood pressure (Pearson correlation coefficient of 0.54 +/− 0.15), dP/dt_max_ (Pearson correlation coefficient of 0.51 +/− 0.18), and stroke volume (Pearson correlation coefficient of 0.54 +/− 0.17). Conclusion: A collection of synchronized, heart-driven signals, including BCGs, ECGs, PPGs, and blood pressure waveforms, was acquired and made publicly available. An initial study indicated that bed-based ballistocardiography can be used to track beat-to-beat changes in systolic blood pressure and stroke volume. Significance: To the best of the authors’ knowledge, no other database that includes time-aligned ECG, PPG, BCG, and continuous blood pressure data is available to the public. This dataset could be used by other researchers for algorithm testing and development in this fast-growing field of health assessment, without requiring these individuals to invest considerable time and resources into hardware development and data collection.

## 1. Introduction

Ballistocardiogram (BCG) systems have the potential to track information such as cardiac beat-to-beat intervals (instantaneous pulse rate) [1], heartrate variability (HRV) features [2], sleep quality [3,4], heart contractility [5], and even blood pressure [6,7]. Given that a BCG is created by the pumping action of the heart, it contains cardiovascular information complementary to other cardiac signals, making it sensible to extract additional information when used in combination with other signals (e.g., a BCG plus a photoplethysmogram (PPG), or a BCG plus an electrocardiogram (ECG)) [8].

**Figure 1:**
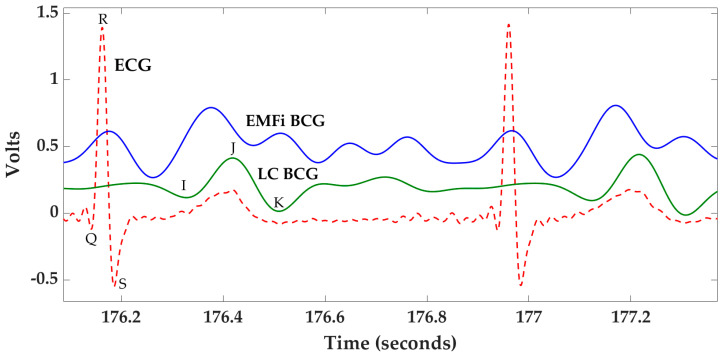
Two sample ballistocardiograms (BCGs) acquired with a bed system using electromechanical film and load cell sensing technologies along with a time-aligned electrocardiogram. The BCG signals have been scaled/shifted vertically. ECG: electrocardiogram; LC BCG: load cell BCG; EMFi: electromechanical film.

A BCG is a representation of the body’s recoil response to the forces created by the heart as it pumps blood into the vascular system [9]. An acquired BCG is thus a representation of a more general three-dimensional signal that contains vector components with axial directions named with respect to the body—front-to-back, head-to-foot, and side-to-side [10]. Different methods to gather BCGs exist, including wearable systems, weighing scales, chair-based systems, and bed-based systems. Although somewhat intrusive, wearable systems, which provide continuous measurements, have been investigated for this purpose [11,12]. Weighing scales, which measure the head-to-foot axial component, are possibly the most researched methodology [6,13,14,15]. However, these systems do not have an important property of other BCG systems—the ability to monitor cardiovascular data unobtrusively without effort from the user. Truly non-unobtrusive systems include chair-, bed-, video-, or radar-based systems [7,16,17,18,19,20,21,22,23]. Of these, our team has experience with bed-based systems, and two sample BCGs acquired with a bed system that employs two different sensing technologies (electromechanical films (EMFis) and load cells) are illustrated in Figure 1 along with a time-aligned ECG. The main BCG features (I, J, and K) are annotated for the load cell BCG (LC BCG).

Often, when new BCG acquisition systems and the related processing algorithms are created and assessed, ground-truth cardiopulmonary data are collected to affirm hypotheses based upon these BCGs; however, complete datasets are rarely illustrated in the resulting publications, much less provided to the reader for follow-on analysis. Not having access to these datasets makes it difficult for other researchers to test new methods or theories related to the original publication (e.g., when seeking to compare the effectiveness of BCG peak-detection algorithms [1]). When such datasets are fully described in a publication and made available (along with code when applicable), comparisons become possible, and significant time can be saved. For example, if a group wants to test a new BCG heartbeat detection algorithm without having access to a pre-existing dataset, they must first invest considerable time into hardware design, development, and verification prior to data collection and algorithm testing.

Our team at Kansas State University has created a bed-based BCG system designed to monitor and quantify the sleep of children who reside at Heartspring, a residential and day school facility in Wichita, KS dedicated to helping children with specialized needs and severe disabilities, including children who are non-verbal and who have disorders across the autism spectrum [24]. The goal of this paper, as a follow-on to this prior work, is two-fold: (1) to outline the bed system in detail, and (2) to provide a dataset to the research community that includes ballistocardiographic signals monitored by the bed system—signals that are synchronized with more traditional heart-related signals, where these data originate from adult participants with a variety of ages and health conditions.

By making this dataset publicly available, this team hopes that accelerated progress can be made in the field of ballistocardiography toward the following:

1. Improved heartbeat detection algorithms;
2. Feasibility assessments related to bed-BCG-based blood pressure tracking;
3. A better understanding of the influence of sensor location and type on BCG morphology;
4. Enhanced motion detection/mitigation algorithms.

The idea of continuously monitoring blood pressure using noninvasive means has become a popular topic in recent years [7,25,26,27,28]. Pulse arrival time (PAT), meaning the time delay between the electrical activation of the heart and the arrival of a commensurate pulse wave at a distal point, has shown promise for tracking changes in blood pressure (see [25] or [29] for an overview of the relationship between PAT and blood pressure). Therefore, in addition to providing the dataset, this paper presents an initial analysis investigating the ability of the bed system to track beat-to-beat changes in blood pressure and associated cardiovascular parameters using BCG-related parameters in comparison with the better-known PAT model.

## 2. Materials and Methods

### 2.1. Bed-Based Ballistocardiography

**Figure 2:**
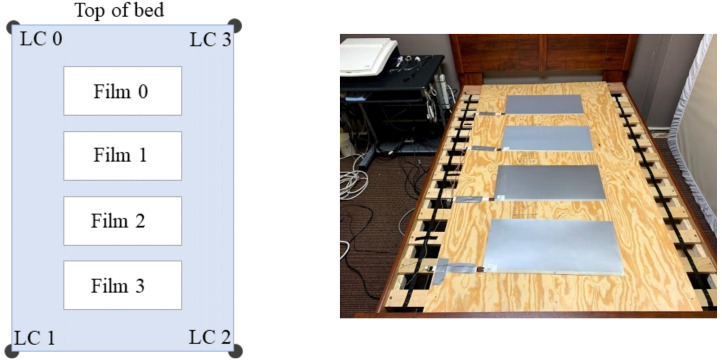
Approximate EMFi and load cell locations (not drawn to scale) (**left**) and the actual film locations (**right**), where the mattress has been removed and is leaning on the wall next to the bed.

**Figure 3:**
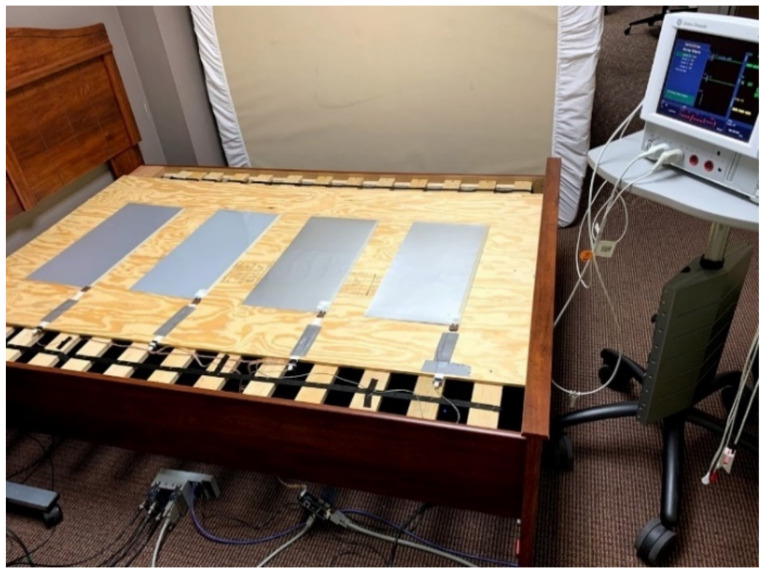
Image of the bed system from another angle, accompanied by the CardioCap 5 patient monitor. The analog conditioning and National Instruments data collection hardware can be seen under the bed.

An early description of, and motivation behind, the design of the bed system used in this study can be found in [24]. The bed system has been used to compare BCG J-peak detection algorithms [1], and it was used to investigate the relationship between sleep and daytime behavior and cognitive function in severely disabled children with autism who reside at Heartspring [30]. Four electromechanical films (EMFit; L series; 300 mm × 580 mm—“EMFi” sensors) and four load cells (TE Connectivity Measurement Specialties FX1901-0001-0200-L—“LC” sensors) acquire a participant’s BCG. Most of the children at Heartspring are in the lower-functioning portion of the autism spectrum, and it was unclear if a given child would sleep in a standard resting position (e.g., supine or prone). Therefore, sensors were placed to encompass a large portion of the bed so as to increase the likelihood of acquiring quality BCGs regardless of sleeping position; the electromechanical films were placed in a single, linear column underneath the mattress, and four load cells were positioned under the respective corner bedposts (see Figure 2 and Figure 3).

The analog signals from each film sensor were amplified and bandpass-filtered between 0.3 and 24 Hz, whereas the analog signals from each load cell were amplified and bandpass filtered between 0.05 and 35 Hz. Two amplification levels were employed for load cell signals: one level to accommodate large signals due to subject movement for estimating center of position (COP) and one level to accommodate smaller, more sensitive BCG signals related to cardiac activity. A computer running LabVIEW version 14.01 managed a National Instruments (NI) 9184 Ethernet chassis housing two NI 9220 analog input modules, which were configured to synchronously sample each of the 12 EMFi and LC analog signals at a sampling rate of 1 kHz. No additional software filtering was applied in the virtual instrument before these data were saved to files.

### 2.2. ECG, PPG, and Continuous Blood Pressure Waveforms

**Figure 4:**
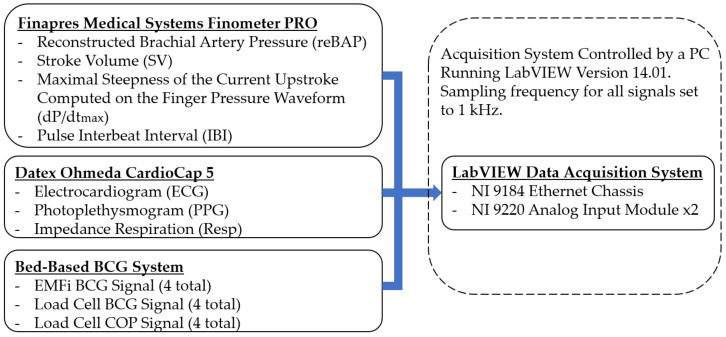
Signal management and acquisition. COP: center of position.

The aggregate signal collection acquired with this bed-based system and the accompanying external equipment is depicted in Figure 4. A GE Datex Ohmeda CardioCap 5 vital signs monitor was used to gather three-lead ECGs (bandpass filtered between 0.5 and 40 Hz), an estimated heart rate (HR), impedance respiration signals, and finger PPGs. Analog output signals from the CardioCap unit delayed at most by 15 ms relative to the bed sensor data were routed to differential input channels of the same NI 9220 analog input module that collected the BCG data, meaning these signals were time-aligned with, and sampled at the same 1 kHz rate as, the BCGs [31]. A Finapres Medical Systems Finometer PRO was used to gather additional cardiovascular information. The Finometer PRO uses tonometric principles to continuously monitor an individual’s arterial blood pressure via a small cuff placed around their finger, and then it derives cardiovascular parameters from that blood pressure signal on a beat-by-beat basis. This system is non-invasive and requires an initial calibration from a brachial artery blood pressure cuff. Four analog output channels from the Finometer PRO were interfaced to a second NI 9220 analog input module connected to the same NI 9184 Ethernet chassis. The analog signals provided by the Finometer PRO were reconstructed brachial artery pressure (reBAP), stroke volume (SV), maximum steepness of the current finger pressure waveform (dP/dt_max_), and the interbeat interval (IBI), with stroke volume calculated using the ModelFlow method after correcting for age, sex, weight, and height.

### 2.3. Data Collection and Shared Database Structure

**Figure 5:**
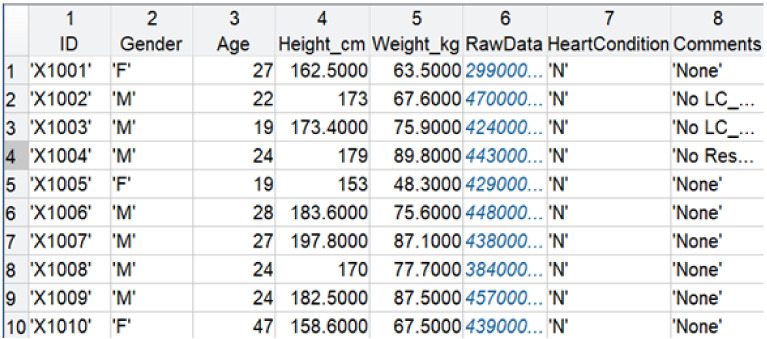
Bed system database excerpt for ten participants.

**Figure 6:**
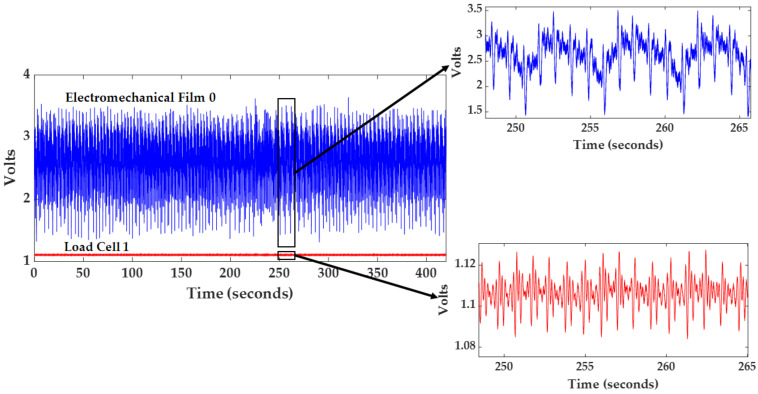
Representative raw signals collected from participant X1003.

Data were collected from 40 participants (17 male) under Kansas State University IRB protocol #9386. Participant demographics are detailed in Table 1. Participant data were collated in a MATLAB table, Bed_System_Database, to simplify further processing. The fields in the MATLAB table include Participant ID, Gender, Age, Height_cm, Weight_kg, RawData, HeartCondition, and Comments. Prior to data collection, each participant was asked in a survey, “Have you ever been diagnosed with any cardiac anomalies or diseases such as atrial fibrillation, arrhythmia, or any other heart conditions? If yes, please list here.” Participant responses to this survey question are included in the field HeartCondition. A HeartCondition of ‘N’ means that the participant did not list any heart conditions on the survey. An image of the MATLAB table, with data visible for ten participants, is illustrated in Figure 5. Note that, during this process, the raw data were de-identified, and unique IDs were assigned. For each participant, the data structure of the RawData field was configured with twenty fields for each acquired signal (PPG, Resp, HR, ECG, Film0, Film1, Film2, Film3, LC_COP0, LC_BCG0, LC_COP1, LC_BCG1, LC_COP2, LC_BCG2, LC_COP3, LC_BCG3, reBAP, IBI, SV, and dp_dt). Illustrative raw EMFi and load cell signals collected from one participant are displayed in Figure 6. Participant ages ranged from 18 to 65 years, and their body mass indices (BMIs) ranged from 18 to 48 kg/m^2^. In total, over 4.5 h of data were collected. Four of the participants indicated that they had some form of past or current cardiovascular-related condition: hypertension, supraventricular tachycardia (addressed by cardiac ablation), atrial fibrillation, and coronary artery disease. These diagnoses are contained in the MATLAB database table (HeartCondition field). For two of the participants, load cell 0 did not have good contact with the bed frame and thus did not collect meaningful BCGs. An additional field, Comments, was added to the database to include such information.

### 2.4. Signal Preprocessing

In addition to introducing the database, this paper presents the results of an initial investigation that looked into the connection between BCG and blood pressure related parameters. Prior to this analysis, the signals were preprocessed using MATLAB version R2019a. BCGs were bandpass filtered between 1 and 10 Hz to reduce noise and to minimize the contribution of respiration components. PPGs and reBAPs were lowpass filtered with a cutoff frequency of 10 Hz. ECGs were bandpass filtered between 1 and 40 Hz. Because the reBAP signal from the Finometer is a reconstructed signal, it has an overall delay of 1 s. This delay was removed prior to analysis. Stroke volume (SV) and dP/dt_max_ signals from the Finometer PRO also experience a 1-s delay coupled with a 1-beat delay. In the preprocessed data, SV and dP/dt_max_ were shifted to compensate for only the 1-s delay. The processed data used for the analysis are included in a separate MATLAB table, “Preprocessed_Database.” Note that, in the preprocessed database, the SV and dP/dt_max_ metrics still have the 1-beat delay. The analysis presented in this paper compensated for the 1-beat delay during the beat-by-beat feature extraction approach described in the following section. Both databases can be found on the IEEE DataPort cloud platform [32].

### 2.5. Initial Analysis—Ballistocardiogram and Blood Pressure Parameter Extraction

**Figure 7:**
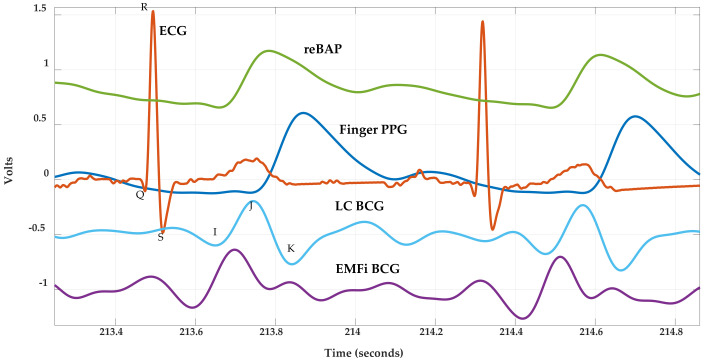
Various cardiopulmonary waveforms and their associated features. The reconstructed brachial artery pressure (reBAP) waveform acquired by a Finapres Finometer PRO^®^ is scaled at 100 mmHg/volt. The BCG signals were scaled/shifted vertically and preprocessed as described in Section 2.4. ECG and PPG data are collected with a GE Datex Ohmeda CardioCap 5 patient monitor.

**Figure 8:**
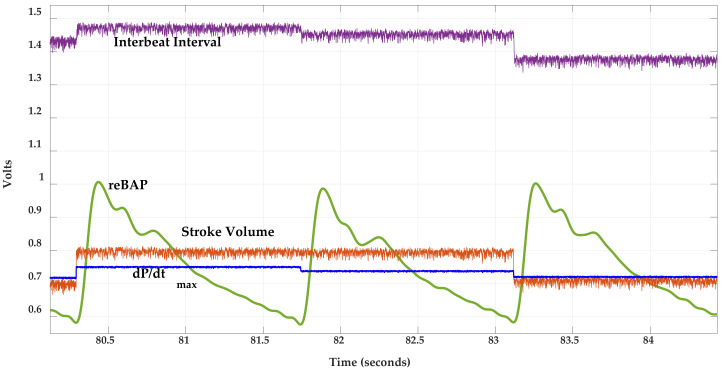
Representative signals collected from the Finapres Finometer PRO^®^. As in Figure 7, the reBAP signal is scaled at 100 mmHg/volt. The interbeat interval, stroke volume, and dP/dt_max_ are scaled at 1000 ms/volt, 100 mL/volt, and 1 mmHg/s/volt, respectively.

Beat-to-beat parameters were extracted from each BCG, PPG, and blood pressure waveform for approximately 100 cardiac cycles, where ECG R-peaks (located using the Pan-Tompkins method [33]) delineated heart beat cycle boundaries. Noisy or motion corrupted data were identified by visual inspection and removed as necessary. Moreover, prior to feature extraction, BCGs measured from film 0, load cell 0, and load cell 3 were inverted owing to their relative position to the body compared with BCGs measured from the other sensors. For each segmented BCG cycle, the most prominent peak that occurred between 100 and 400 ms after the prior ECG R peak was identified as the J peak. The corresponding I and K features were then determined as the closest local minima prior to and after the J peak, respectively. The maximum point of the first derivative (estimated using the MATLAB function, diff()) for the rising edge of a PPG cycle was used to compute pulse arrival time (PAT) [25]. The maximum and minimum points of a reBAP cycle provided systolic and diastolic pressures. SV and dP/dt_max_ metrics were averaged over one cardiac cycle and then shifted to compensate for the 1-beat delay. Figure 7 and Figure 8 illustrate the various waveforms and their associated features.

### 2.6. Relating ECG, PPG, and BCG Extracted Features to Cardiovascular Parameters

Univariate and multivariate analyses were performed to link ECG, PPG, and BCG features to cardiovascular parameters. Table 2 lists and describes these beat-to-beat parameters, and Table 3 lists the predictive variables and the associated desired responses for the multivariate analyses. The univariate model to estimate systolic pressure (SP) from PAT during cardiac cycle, *i*, is expressed in Equation (1), where the scalar coefficients, *k_0_* to *k_n_*, are determined for each participant. The more general multivariate regression model is expressed in Equation (2), where *Response_i_* represents a general response parameter (see Table 3, right column) during the cardiac cycle, *i*. The *m_n_* values are scalar coefficients (also determined for each participant), and *BCG_pni_* represents the *n*^th^ BCG predictor, *p* (see Table 3, left column) during the cardiac cycle, *i*. The relationship between PAT and systolic blood pressure is inversely proportional, thus PAT estimates are often either inverted or log-transformed prior to model fitting [25]. For the analysis presented here, the PAT estimates were log-transformed (the *ln* () in Equation (1) is a natural logarithm). Given that the BCG timing parameters have been linked to blood pressure according to similar principles [5,6], those timing estimates were also log-transformed prior to model fitting. To smooth out any jitter, a five-wide moving average filter (employing the current value and the previous four estimates) was applied to the predictor and response variables prior to solving for the coefficients.

(1) $$SP_{i}=k_{0}+\;k_{1}\;*\;ln\left(PAT_{i}\right)$$

(2) $$Response_{i}=\;m_{0}+\;m_{1}\;*\;BCG_{p1i}+\;\ldots \;+\;m_{n}\;*\;BCG_{pni}$$

For the univariate and multivariate approaches, least squares linear regression approaches were employed to fit each model to the corresponding dataset. The BCG parameter(s) were treated as the predictor variable(s)—see Table 3, left column. Cardiovascular parameters were treated as response variables—see Table 3, right column. The coefficients of the linear model were then used to compute estimated cardiovascular values (e.g., beat-to-beat systolic blood pressures). The Pearson correlation coefficient was computed between the estimated and true beat-to-beat values for each participant using approximately 100 heartbeat intervals.

## 3. Results

The metrics in Table 4 were derived from the preprocessed dataset. Average heart rate varied considerably between participants, ranging from 42 to 92 beats per minute. The systolic pressure range (max−min) varied from 11 to 46 mmHg, and the diastolic pressure range varied from 5 to 27 mmHg.

**Figure 9:**
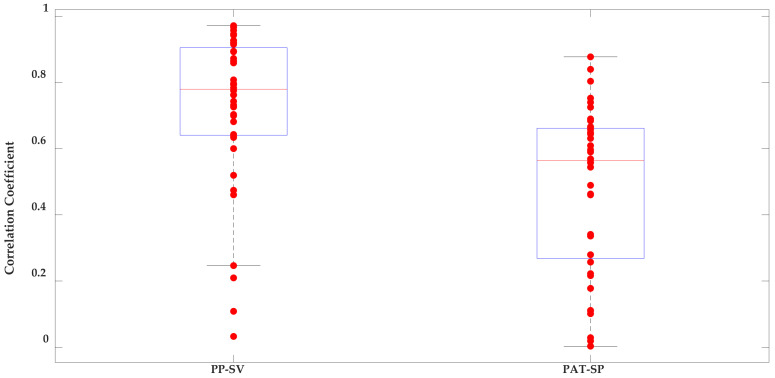
Boxplots of the correlation coefficients for the pulse pressure (PP)–stroke volume (SV) and pulse arrival time (PAT)–systolic pressure (SP) predictor–response relationships.

Table 5 presents the correlation coefficients for two predictor–response combinations. Pulse pressure, which is known to be well-correlated to SV [34], did offer a high average correlation coefficient of 0.72 +/− 0.24. The average correlation coefficient for PAT-estimated systolic blood pressure was 0.48 +/− 0.25. Boxplots illustrating the correlation coefficients for all 40 participants can be seen in Figure 9.

**Figure 10:**
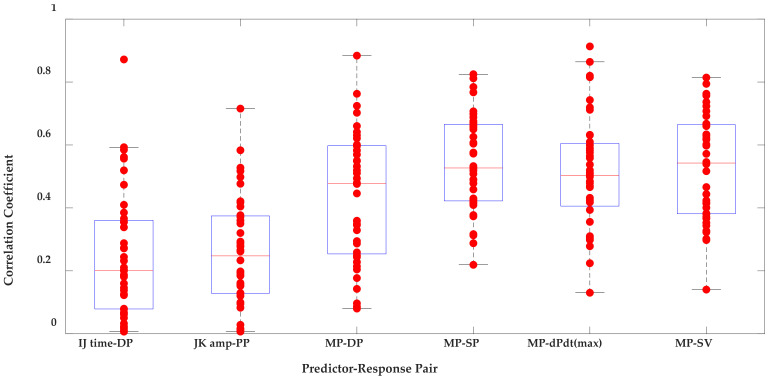
Boxplots of the correlation coefficients for the predictor–response pairs described in Table 3. using the BCGs measured from Film 0. MP: multiple parameters (IJ time, IJ amp, JK time, and JK amp).

Table 6 presents univariate and multivariate model results for each sensor. The estimated cardiovascular parameters using single BCG predictors (the first two columns in Table 6) did not correlate well with the paired cardiovascular response parameters; the average correlation coefficients ranged from 0.17 to 0.29. The bed system did show higher correlation coefficients when multiple BCG predictors were considered (columns 3 through 6 in Table 6). For Film 0, average correlation coefficients of 0.54 (*p* < 0.05 for 39 out of 40 participants), 0.51 (*p* < 0.05 for 40 out of 40 participants), and 0.54 (*p* < 0.05 for 35 out of 40 participants) were found when estimating SP, dP/dt_max_, and SV, respectively. Figure 10 presents these results in the form of boxplots.

## 4. Discussion

The average PAT correlation coefficient (0.48 +/− 0.25) is slightly lower compared to other coefficients reported in the literature (0.66 +/− 0.15 [35] (fifteen young and healthy volunteers); 0.59 [29]). Three participant datasets had a poor-quality or weak-amplitude PPG (X1019, X1037, and X1046). Removing these three datasets from the analysis improves the mean PAT correlation coefficient to 0.52 +/− 0.22. Given that the bed system records BCGs that represent a superposition of the dorsoventral and head-to-foot axes, it seems reasonable to not expect the same results as seen in other BCG-based blood pressure tracking publications that predominately measure the head-to-foot axial BCG component (i.e., standing–BCG measurement systems). In one publication where bed-based BCG features demonstrated a high correlation with blood pressure, the authors did not consider continuous or beat-to-beat pressures, but instead investigated blood pressure values measured from a cuff-based system [7]. The dataset presented here makes it possible to perform a multitude of studies investigating various aspects of bed-based ballistocardiography. This initial analysis addressed cardiovascular metrics for 40 participants of varying ages and body types.

### Limitations

Each participant laid in the same position (supine) with their head close to Film 0. However, the exact position of their heart relative to each sensor varied from one participant to the next, owing in part to variations in participant height. While this lack of exact positioning may be a form of noise in the data, the dataset does represent the results of ecologically valid measurements. The same mattress type was used for all participants. Future studies that contribute signals to this database will consider mattresses of different types as well as different resting positions.

In other studies that attempted to track blood pressure using features extracted from BCGs, protocols to modulate blood pressure were often conducted (e.g., mental arithmetic, breath holding, and exercise; a list of commonly used intervention techniques is given in [25]). However, our shared dataset includes only endogenous changes in blood pressure observed as the participants laid on the bed for this study. Still, these changes were at times considerable—one participant had a systolic pressure range of 46 mmHg for the window of data analyzed. The average cardiovascular parameter ranges can be seen in Table 4. Finally, most of the participants had no history of cardiovascular disease. As the database is expanded, more participants with prior or current cardiovascular conditions will be recruited.

## 5. Conclusions

BCG acquisition systems have regained popularity in recent years thanks to their ability to acquire cardiopulmonary information without requiring user intervention. However, such datasets as acquired and employed by researchers to present new algorithms or test new theories are not typically made available to the research community. This paper described a bed-based system to acquire several heart-driven signals, with a goal to provide a diverse set of heart-related biomedical signals, including ballistocardiograms, to research teams that wish to develop and test novel algorithms without the need to invest time and resources toward physical bed hardware and data acquisition equipment. Further, the results of an initial investigation that assessed the ability of a bed-based BCG to monitor changes in cardiovascular function were also presented.

## Figures and Tables

**Table 1:** Participant demographics (40 total, 17 male). BMI: body mass index.

Characteristic	Mean +/− Stdev
Age (years)	34 +/− 15
Weight (kg)	76 +/− 18
Height (cm)	171 +/− 11
BMI (kg/m^2^)	26 +/− 5.7

**Table 2:** Beat-to-beat electrocardiogram (ECG), photoplethysmogram (PPG), ballistocardiogram (BCG), and cardiovascular parameters.

Parameter	Description
PAT	Time delay between the PPG maximum first derivative and ECG R peak
IJ time	Time delay between the BCG J and I peaks
JK time	Time delay between the BCG K and J peaks
IJ amp	Amplitude difference between the BCG I and J peaks
JK amp	Amplitude difference between the BCG J and K peaks
SP	Systolic blood pressure
DP	Diastolic blood pressure
PP	Pulse pressure (systolic pressure–diastolic pressure)
SV	Stroke volume
dP/dt_max_	Maximal steepness on the upstroke of the finger pressure waveform

**Table 3:** Predictor(s)–response pairs. SV: stroke volume; SP: systolic pressure; DP: diastolic pressure; PP: pulse pressure.

Predictor(s)	Response
Pulse Pressure	SV
PAT *	SP
IJ time *	DP
JK amp	PP
IJ time *, IJ amp, JK time *, JK amp	DP
IJ time *, IJ amp, JK time *, JK amp	SP
IJ time *, IJ amp, JK time *, JK amp	dP/dt_max_
IJ time *, IJ amp, JK time *, JK amp	SV

* Estimates were log-transformed prior to model fitting.

**Table 4:** Cardiovascular variation.

Cardiovascular Response	Avg. Range (Max−Min) (Mean +/− Stdev)
Systolic Pressure (mmHg)	21 +/− 8.6
Diastolic Pressure (mmHg)	13 +/− 5.0
Stroke Volume (ml)	23 +/− 9.9
dP/dt_max_ (mHg/s)	0.31 +/− 0.15

**Table 5:** Correlation coefficients for estimated and true cardiovascular parameters averaged across all participants. PAT: pulse arrival time.

Predictor	Cardiovascular Response	Correlation Coefficient (Mean +/− Stdev)
PP (Pulse Pressure)	Stroke Volume	0.72 +/− 0.24
PAT *	Systolic Pressure	0.48 +/− 0.25

* Estimates were log-transformed prior to model fitting.

**Table 6:** Correlation coefficient (mean +/− stdev.) for each response(s)–predictor pair and each sensor. MP: multiple BCG parameters (IJ time *, JK time *, IJ amp, JK amp). PP: pulse pressure. DP: diastolic pressure. SP: systolic pressure.* Estimates were log-transformed prior to model fitting.

Sensor	IJ time–DP	JK amp–PP	MP–DP	MP–SP	MP–dP/dt_max_	MP–SV
Film 0	0.25 +/− 0.20	0.26 +/− 0.17	0.44 +/− 0.2	0.54 +/− 0.15	0.51 +/− 0.18	0.53 +/− 0.17
Film 1	0.25 +/− 0.19	0.22 +/− 0.17	0.43 +/− 0.21	0.52 +/− 0.16	0.50 +/− 0.13	0.54 +/− 0.15
Film 2	0.22 +/− 0.15	0.23 +/− 0.17	0.44 +/− 0.17	0.49 +/− 0.18	0.48 +/− 0.19	0.52 +/− 0.20
Film 3	0.29 +/− 0.17	0.21 +/− 0.18	0.44 +/− 0.21	0.52 +/− 0.15	0.49 +/− 0.16	0.48 +/− 0.17
Load Cell 0 ^1^	0.20 +/− 0.16	0.17 +/− 0.15	0.44 +/− 0.18	0.51 +/− 0.16	0.50 +/− 0.16	0.52 +/− 0.17
Load Cell 1	0.20 +/− 0.19	0.19 +/− 0.15	0.41 +/− 0.18	0.51 +/− 0.15	0.48 +/− 0.17	0.54 +/− 0.17
Load Cell 2	0.24 +/− 0.16	0.22 +/− 0.15	0.45 +/− 0.18	0.53 +/− 0.14	0.49 +/− 0.16	0.53 +/− 0.14
Load Cell 3	0.23 +/− 0.16	0.23 +/− 0.18	0.40 +/− 0.19	0.50 +/− 0.15	0.49 +/− 0.17	0.54 +/− 0.17

^1^ Data from participants X1002 and X1003 were excluded from the analysis because of poor signal quality.

## Data Availability

The data presented in this study are openly available in IEEE DataPort at doi:10.21227/77hc-py84, reference number [32].
